# Supplementary material for: Empirical methods for controlling false positives and estimating confidence in ChIP-Seq peaks
Source: BMC Bioinformatics. 2008 Dec 5;9:523. doi: 10.1186/1471-2105-9-523 (PMC2628906; doi:10.1186/1471-2105-9-523)
Supplement: Additional File 1 — A variety of html documents from the USeq web site detailing the available applications, their best usage, output file type descriptions, command line menus, etc. [file 1471-2105-9-523-S1.zip › USeqUserGuides/JavaDoc.zip!/JavaDoc/edu/utah/seq/analysis/QCSeqs.html]

QCSeqs


|  |  |  |
| --- | --- | --- |
| **Overview** \| **Package** \| **Class** \| **Use** \| **Tree** \| **Deprecated** \| **Index** \| **Help** | |  |
| **PREV CLASS**   **NEXT CLASS** | **FRAMES**    **NO FRAMES**     **All Classes** |  |
| SUMMARY: NESTED \| FIELD \| CONSTR \| METHOD | DETAIL: FIELD \| CONSTR \| METHOD |  |


---


## edu.utah.seq.analysis Class QCSeqs

```
java.lang.Object
  edu.utah.seq.analysis.QCSeqs
```

---

``` public class QCSeqs extends java.lang.Object ```

Takes PointData and generates sliding window average scores.
The Poisson probability is based on a hit count so if your Point Data isn't such then use with caution.

---

**Constructor Summary**

`QCSeqs(java.lang.String[] args)`


| **Method Summary** | |
| --- | --- |
| `void` | `correlate()` |
| `double` | `correlate(java.util.HashMap<java.lang.String,edu.utah.seq.analysis.QCSeqs.ChromWindowScores> first, java.util.HashMap<java.lang.String,edu.utah.seq.analysis.QCSeqs.ChromWindowScores> second)` |
| `edu.utah.seq.analysis.QCSeqs.ChromWindowScores[]` | `fetchChromSpecificWindowScores(java.lang.String chrom)` |
| `void` | `loadMissingChromosomes()` |
| `static void` | `main(java.lang.String[] args)` |
| `int` | `maxPosition(java.lang.String chrom)` |
| `void` | `mergeAndSavePointData()` |
| `static void` | `printDocs()` |
| `void` | `processArgs(java.lang.String[] args)`             This method will process each argument and assign new varibles |
| `float` | `sumWindowScores(int startBp, int stopBp, edu.utah.seq.analysis.QCSeqs.ChromWindowScores pd)` |
| `void` | `windowScorePointData()`             Calculates window averages for merged PointData. |

**Methods inherited from class java.lang.Object**

`equals, getClass, hashCode, notify, notifyAll, toString, wait, wait, wait`

**Constructor Detail**

### QCSeqs

```
public QCSeqs(java.lang.String[] args)
```


**Method Detail**

### correlate

```
public void correlate()
```

---


### correlate

```
public double correlate(java.util.HashMap<java.lang.String,edu.utah.seq.analysis.QCSeqs.ChromWindowScores> first,
                        java.util.HashMap<java.lang.String,edu.utah.seq.analysis.QCSeqs.ChromWindowScores> second)
```

---


### loadMissingChromosomes

```
public void loadMissingChromosomes()
```

---


### windowScorePointData

```
public void windowScorePointData()
```

:   Calculates window averages for merged PointData.

---


### sumWindowScores

```
public float sumWindowScores(int startBp,
                             int stopBp,
                             edu.utah.seq.analysis.QCSeqs.ChromWindowScores pd)
```

---


### fetchChromSpecificWindowScores

```
public edu.utah.seq.analysis.QCSeqs.ChromWindowScores[] fetchChromSpecificWindowScores(java.lang.String chrom)
```

---


### maxPosition

```
public int maxPosition(java.lang.String chrom)
```

---


### mergeAndSavePointData

```
public void mergeAndSavePointData()
```

---


### main

```
public static void main(java.lang.String[] args)
```

---


### processArgs

```
public void processArgs(java.lang.String[] args)
```

:   This method will process each argument and assign new varibles

---


### printDocs

```
public static void printDocs()
```


---


|  |  |  |
| --- | --- | --- |
| **Overview** \| **Package** \| **Class** \| **Use** \| **Tree** \| **Deprecated** \| **Index** \| **Help** | |  |
| **PREV CLASS**   **NEXT CLASS** | **FRAMES**    **NO FRAMES**     **All Classes** |  |
| SUMMARY: NESTED \| FIELD \| CONSTR \| METHOD | DETAIL: FIELD \| CONSTR \| METHOD |  |


---
